# Strain and strain rate echocardiographic imaging predict occurrence of atrial fibrillation in post-coronary artery bypass grafting patients

**DOI:** 10.1186/s43044-021-00188-z

**Published:** 2021-07-03

**Authors:** Gomaa Abdelrazek, Kareem Mandour, Mohammad Osama, Khaled Elkhashab

**Affiliations:** 1 Cardiology Department, Faculty of Medicine, Fayoum University, Faiyum, Egypt; 2 National Heart Institute, Cairo, Egypt

**Keywords:** Left atrial dysfunction, Left atrial strain, Postoperative atrial fibrillation, Speckle tracking

## Abstract

**Background:**

Atrial fibrillation (AF) occurs very frequently after coronary artery bypass grafting (CABG); it occurs in about 20–edictors can be used for the dedicatio40% of patients. It is associated with several adverse events. This study aimed to extrapolate a predictor for postoperative atrial fibrillation (POAF) occurrence which is reproducible and simple to be a part of routine echocardiography screening before CABG. This study included 89 patients scheduled for isolated coronary artery bypass surgery. History, clinical examination, and complete 2D echocardiography with LA speckle tracking analysis were done preoperatively. Patients were then followed up post-surgery for incidence of AF till discharge from the hospital. The patients were divided into 2 groups according to POAF occurrence.

**Results:**

Patients who developed postoperative AF had older age (*P* = 0.0032) and longer hospital stay (*P* = 0.021) and higher stroke incidence but statistically non-significant (14.3% vs 3.3%). The POAF patients showed less peak atrial longitudinal strain (PALS) value than non-POAF patients. The left atrial strain rate values showed a significant difference with the lower left atrial systolic strain rate and less negative (higher) early diastolic strain rate and late diastolic strain rate. After multivariate logistic regression analysis, the independent predictors for POAF were PALS (OR 0.770, 95% CI 0.627–0.946), late LA diastolic strain rate (LASRa) (OR 3.476, 95% CI 1.207–12.186), and age (OR 1.181, 95% CI 1.011–1.379).

**Conclusion:**

Preoperative LA global strain assessed by 2D speckle tracking analysis could be helpful as a predictor for AF post-CABG surgery, and identification of these patients may reduce its morbidity and mortality. The study suggested PALS value less than 29.8 to be a predictor for the occurrence of POAF.

## Background

Atrial fibrillation is a frequent complication after coronary artery bypass grafting surgery; it occurs in about 20 to 40% of patients. AF can cause hemodynamic compromise, thromboembolic complications, and prolonged hospitalization [1].

So, patients with a higher risk of postoperative AF (POAF) occurrence should be identified to develop prophylactic strategies in risky subjects [2]. Also, it is crucial to select patients who will benefit from good prophylactic management or intensive monitoring. Because of the limited understanding of POAF pathophysiology, no effective prophylaxis treatment has been developed yet. Multiple causes as inflammation, oxidative stress, atrial fibrosis, and changes in expression of atrial connexins contribute to the development of pro-arrhythmia [3].

The LA has a significant contribution in cardiac performance. The mechanical LA function has 3 components: (1) a reservoir function during left ventricular systole, (2) a passive conduit for transferring blood from the pulmonary veins to LV during early ventricular diastole, and (3) a booster pump function with an active LA emptying during late ventricular diastole [4].

Tissue Doppler imaging (TDI) and 2D speckle tracking (strain and strain rate) for myocardial deformation are extensively used for the early detection of myocardial dysfunction with high sensitivity [5]. Left atrial enlargement and dysfunction were powerful markers for increased mortality in patients with CAD. There is a lack of literature as regards the assessment of LA function. Unlike the Doppler-derived strain and strain rate, 2DSE is angle non-dependent [6]. Assessment of LA deformation by Doppler-derived strain imaging has been used for LA function evaluation. However, this method has limited reproducibility and angle dependence and is affected by noise artifacts [7].

LA strain was a good prognostic indicator of AF recurrence after cardioversion over a 6-month follow-up, as improvement in PALS was more pronounced in patients who persisted in sinus rhythm than those with AF recurrence [8].

## Methods

This is a prospective observational study that included 89 consecutive patients presenting in the period of May 2019 to August 2020 for elective CABG with normal LV ejection fraction (> 50%) and normal sinus rhythm with no history of prior attacks of AF and no valvular lesions.

Patients were subdivided into 2 groups after surgery:

*Group 1* who did not develop postoperative AF (POAF)

*Group 2* who developed postoperative AF (POAF)

Coronary angiography 4–12 weeks before the surgery was done for all patients. A coronary artery narrowing luminal diameter of ≥70% (≥50% in the left main coronary artery) was considered significant.

Exclusion criteria:

- Patients in need of valvular surgery (repair or replacement) besides CABG (including patients with moderate or higher grades of valvular lesions)
- Patients with rhythms other than normal sinus rhythm
- Patients with a previous history of AF
- History of acute coronary syndrome for less than 30 days
- Patients with right ventricular dysfunction, enlargement, or pulmonary hypertension
- Active inflammatory or infectious diseases, renal dysfunction (serum creatinine > 1.5 mg/dL) or renal failure, COPD, and patients with hyperthyroidism or hypothyroidism

The included patients were subjected to:

- History taking, including age, sex, medications, current symptoms, any other diseases, and comorbidities
- Clinical examination, vital signs (heart rate, blood pressure), class of heart failure (NYHA class), chest pain (Canadian class), body weight measurement, and ECG for documentation of sinus rhythm

Operative and postoperative data:

- Bypass time and cross-clamping time
- Number of bypassed vessels
- Incidence of MI and stroke postoperative
- Inotropic support postoperative
- Postoperative serum potassium
- Duration of hospital stay
- Clinical follow-up data was obtained from inpatient postoperative ICU examinations

Patients were continuously monitored for 1 week after CABG for detection of POAF (any AF episode lasting more than 30 s).

### Standard echocardiography

Baseline 2D echocardiogram with apical 2- and 4-chamber views was done with the patient in the left lateral decubitus by a single trained operator with a probe 2–4 MHz (Philips iE33 machine). The echocardiography was done in the day prior to surgery. Assessment of LV and LA diameters was done according to the current ASE (American Society of Echocardiography) recommendations. LV ejection fraction was measured using the modified biplane Simpson’s method. LA volumes were measured from apical 4- and 2-chamber views using the biplane method. Pulsed-wave Doppler at the mitral valve leaflets tips in an apical 4-chamber view and tissue Doppler velocities of medial and lateral mitral annulus were used for evaluating LV diastolic function. The mitral and aortic valve opening and closure timings were assessed using pulsed-wave Doppler tracings of LV inflow and outflow and tissue Doppler.

### Speckle tracking

Peak atrial longitudinal strain (PALS) was obtained from the average of values observed in all segments of the LA in 4-, 3-, and 2-chamber views.

The graph of global and segmental SR and the numeric data both segmental and global average strain values in the “bulls-eye” format could be obtained.

In our study, the start point for processing software in strain and strain rate measurements was the peak R wave in ECG monitoring. The results of the Mascot trial are consistent with the method used in our study showing that QRS measurements are more accurate than P wave measurements and more reproducible [9].

PALS was calculated at the termination of the reservoir phase on the strain curve (the peak positive curve value at the strain curve).

*Strain rate* curve for each atrial segment in the apical 4-chamber view:

The first negative deflection after R wave on ECG was E-wave (the conduit phase in early diastole).

The second negative deflection after R wave on ECG was A-wave (the LA contractile phase in late diastole).

Positive deflection was S-wave (the reservoir phase during ventricular systole).

*SR E*, *SR A*, and *SR S* were measured at the peak of those three waves, respectively.

To measure strain and strain rate of LA walls, offline analysis was done using Philips Q lab 10 software. The LA wall was divided into six segments from the septal mitral annulus to the lateral mitral annulus in the apical four-chamber view, first and second segments correspond to the inter-atrial septum, third and fourth segments to the roof of the LA, and fifth and sixth segments to the lateral wall. The same software used for the analysis of ventricular function was used as there is still no software for atrial strain assessment.

### Statistics

Data were analyzed using Statistical Program for Social Science (SPSS) version 25 for windows (SPSS Inc., Chicago, IL, USA). Quantitative variables were presented as mean ± standard deviation (SD). Qualitative variables were presented as percentages.

Independent-samples t-test was used when comparing two means of normally distributed variables. The chi-square (X^2^) test was used to compare between two categorical variables. Fisher exact test was used in the place of the chi-square test in 2 by 2 tables.

Multivariate logistic regression was used to predict the value of a variable based on the value of two or more other variables. Receiver operating characteristic (ROC) curve analysis was used to identify optimal cut-off values. *p*-value ≤ 0.05 was considered significant.

### Ethics approval and consent to participate

Study approval was given from the ethical review committee of our Faculty before the study conduction, and every patient was assigned an informed written consent according to the principles of the Local Ethical Committee (Committee reference number: not applicable).

## Results

Patients who developed AF were statistically significantly older than patients who did not develop AF (*P* value = 0.032). There were no statistically significant differences between the 2 groups as regards gender, BMI, hypertension, diabetes, and smoking. The 2 groups showed no statistical significance in NYHA class, Canadian class, serum creatinine levels, heart rate, or the number of coronary arteries affected. Regarding the preoperative medications, there was no statistical significance except for preoperative nitrate administration (Table 1).

**Table 1:** Comparison between the study groups as regards the demographic data, clinical data, and medications

Demographic data	No POAF	POAF	*P*-value
Count	61	28	
**Age (years)**	55.0 ± 6.4	58.7 ± 9.2	0.032
**BMI (kg/m**^**2**^**)**	31.5 ± 4.1	33.0 ± 3.1	0.093
**Risk factors**			
Male gender	40 (65.6%)	18 (64.3%)	0.906
HTN	38 (62.3%)	20 (71.4%)	0.401
DM	28 (45.9%)	18 (64.3%)	0.107
Smoking	32 (52.5%)	16 (57.1%)	0.681
**Clinical data**			
**NYHA class**			
Class 1	42 (68.9%)	19 (67.9%)	0.925
Class 2	19 (31.1%)	9 (32.1%)	
**CCS class**			
Class 1	23 (37.7%)	6 (21.4%)	0.723
Class 2	29 (47.5%)	21 (75%)	
Class 3	9 (14.8%)	1 (3.6%)	
**Heart rate (beat/min)**	76.4 ± 10.6	75.5 ± 10.8	0.708
**Creatinine (mg/dL)**	0.90 ± 0.25	0.94 ± 0.26	0.726
**CAD type**			
Single vessel disease	4 (6.6%)	0 (0%)	0.615
Two-vessel disease	12 (19.7%)	11 (39.3%)	
Multi-vessel disease	45 (73.7%)	17 (60.7%)	
**Medication**			
Aspirin	61 (100%)	28 (100%)	1.000
Statins	46 (75.4%)	19 (67.9%)	0.456
Nitrates	56 (91.8%)	16 (57.1%)	< 0.001
Beta blockers	50 (82%)	20 (71.4%)	0.260
CCB	4 (6.6%)	2 (7.1%)	1.000
Digoxin	0 (0%)	1 (3.6%)	0.315
Diuretic	14 (23%)	3 (10.7%)	0.173
ACEI or ARB	46 (75.4%)	18 (64.3%)	0.278

The operative data were nearly similar with no statistical significance between the 2 groups The postoperative data showed no statistical significance except for the hospital stay, where the no POAF group showed a longer hospital stay (*P* value = 0.021) (Table 2).

**Table 2:** Comparison between the study groups as regards the operative and postoperative data

Operative data	No POAF	POAF	*P*-value
Count	61	28	
**Cross-clamp (min)**	72.4 ± 17.7	65.2 ± 12.6	0.056
**Bypass time (min)**	101.9 ± 19.7	94.3 ± 10.6	0.060
**Number of grafts**			
One	4 (6.6%)	0 (0%)	0.388
Two	23 (37.7%)	11 (39.3%)	
Three or more	34 (55.7%)	17 (60.7%)	
**Postoperative data**			
**K level (mmol/L)**	4.0 ± 0.5	4.0 ± 0.5	0.984
**Cardiac supports**			
Inotropes	42 (68.9%)	21 (75%)	0.554
IABP	3 (4.9%)	4 (14.3%)	0.200
**Postoperative complications**			
MI	8 (13.1%)	3 (10.7%)	1.000
Stroke	2 (3.3%)	4 (14.3%)	0.075
**Hospital stay (days)**	10.4 ± 2.3	11.5 ± 1.8	0.021

The preoperative echocardiographic data showed several significant differences. The POAF patients had a lower mitral E velocity at mitral Doppler inflow measurements, a lower E/A ratio, and a longer mitral E DT. The tissue Doppler measurements showed a statistically lower e’ value in the POAF group. Two-dimensional speckle tracking drove peak atrial longitudinal strain (PALS) showed a lower value in the POAF group. The strain rate values showed a statistical difference with a lower systolic strain rate and higher (less negative) early and late diastolic strain rates (Table 3).

**Table 3:** Comparison between the study groups as regards the echocardiographic measurements

Echocardiographic data	No POAF	POAF	*P*-value
	Mean ± SD	Mean ± SD	
LVEDD (mm)	52.8 ± 4.8	51.7 ± 5.9	0.352
LVESD (mm)	34.5 ± 4.7	34.2 ± 4.9	0.785
LVEF (%)	61.8 ± 7.9	61.5 ± 5.3	0.879
LA diameter (mm)	36.3 ± 3.3	37.1 ± 2.5	0.219
Mitral E velocity (cm/s)	70.7 ± 17.7	55.8 ± 10.6	< 0.001
Mitral A velocity (cm/s)	68.7 ± 18.3	68.6 ± 17.4	0.988
Mitral E/A	1.07 ± 0.35	0.89 ± 0.38	0.025
Mitral E' velocity (cm)	7.1 ± 2.4	5.5 ± 2.1	0.003
Mitral A' velocity (cm)	8.4 ± 2.2	8.9 ± 1.4	0.310
Mitral S' velocity (cm)	7.0 ± 1.6	6.8 ± 1.4	0.373
Mitral E/E'	10.6 ± 2.8	11.2 ± 3.2	0.413
Mitral E deceleration (ms)	201.6 ± 38.9	236.0 ± 27.8	< 0.001
PALS (%)	32.9 ± 5.9	25.6 ± 6.1	< 0.001
LASRs (s^−1^)	1.90 ± 0.71	1.27 ± 0.56	< 0.001
LASRe (s^−1^)	−1.69 ± 0.60	−1.00 ± 0.45	< 0.001
LASRa (s^−1^)	−2.21 ± 0.59	−1.21 ± 0.48	< 0.001

**Fig. 1:**
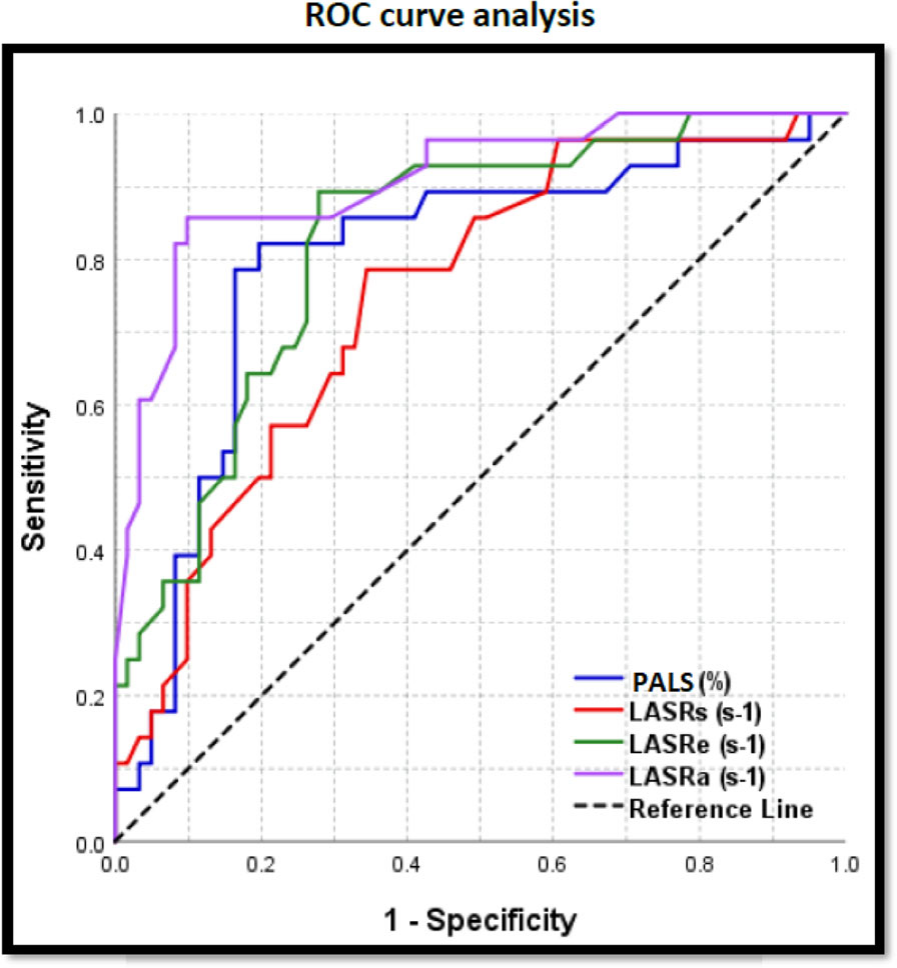
ROC curve analysis

**Fig. 2:**
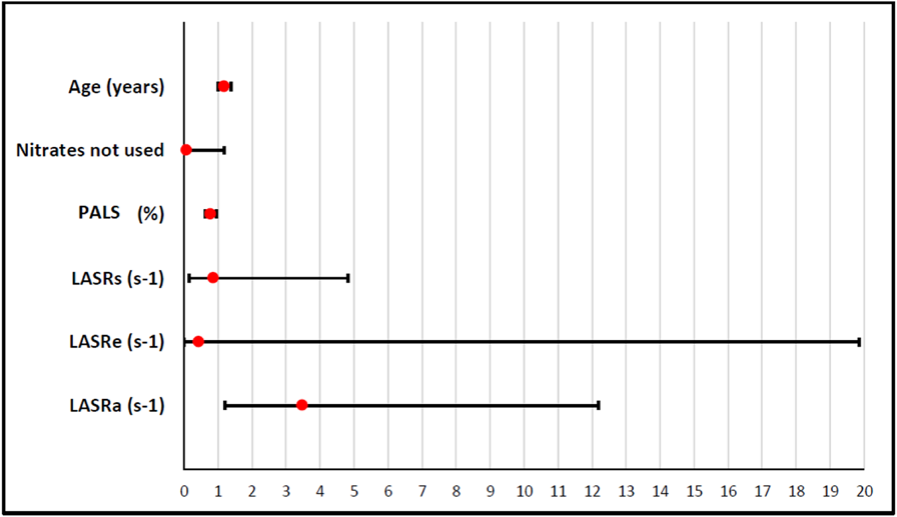
Multivariate regression analysis for incidence of POAF

For the cut-off value of LA strain and strain rate for POAF and ROC curve analysis and multivariate analysis, the only predictor variables for the POAF occurrence were age, PALS, and late atrial strain rate, and PALS of less than 29.8 is the cut-off value for the occurrence of POAF (Tables 4 and 5 and Figs. 1 and 2).

**Table 4:** The cut-off value of LA strain and strain rate for POAF and ROC curve analysis

Cut-off value	SN % (95% CI)	SP % (95% CI)	PPV % (95% CI)	NPV % (95% CI)	Accuracy % (95% CI)	AUC (95% CI)	*P*-value
PALS (%) ≤ 29.8	82.1% (63.1–93.9)	80.3% (68.2–89.4)	65.7% (47.8–80.9)	90.7% (79.7–96.9)	80.9% (71.2–88.5)	0.800 (0.667–0.884)	< 0.001
LASRs (s^−1^) ≤ 1.6	78.6% (59.1–91.7)	65.6% (52.3–77.3)	51.2% (35.5–66.7)	87% (73.7–95.1)	69.7% (59.0–79.0)	0.749 (0.622–0.838)	< 0.001
LASRe (s^−1^) ≤ −1.36	82.1% (63.1–93.9)	73.8% (60.9–84.2)	59% (42.1–74.4)	90% (78.2–96.7)	76.4% (66.2–84.8)	0.824 (0.710–0.896)	< 0.001
LASRa (s^−1^) ≤ −1.67	85.7% (67.3–96.0)	90.2% (79.8–96.3)	80% (61.4–92.3)	93.2% (83.5–98.1)	88.8% (80.3–94.5)	0.908 (0.810–0.957)	< 0.001

*ROC curve* receiver operating characteristic curve, *SN* sensitivity, *SP* specificity, *PPV* positive predictive value, *NPV* negative predictive value, *AUC* area under the curve, *95% CI* 95% confidence interval

**Table 5:** Multivariate regression analysis for incidence of POAF

Variable	Adjusted OR	95% confidence interval for OR		*P*-value
		Lower bound	Upper bound	
**Age (years)**	1.181	1.011	1.379	0.036
**Nitrates not used**	0.069	0.004	1.170	0.064
**PALS (%)**	0.770	0.627	0.946	0.013
**LASRs (s**^**−1**^**)**	0.855	0.151	4.829	0.859
**LASRe (s**^**−1**^**)**	0.434	0.009	19.856	0.669
**LASRa (s**^**−1**^**)**	3.476	1.207	12.186	0.022

## Discussion

Left atrial contractile dysfunction has been observed in CAD patients and it has been considered as a potential risk factor for the occurrence of AF [10].

Strain rate echocardiography (STE) is a feasible and reproducible tool for the objective evaluation of cardiac wall deformation [7]. STE has multiple advantages over classic Doppler-derived parameters, as it is angle non-dependent and has less noise effects, dropouts, and artifacts. It is frequently used for atrial function evaluation [11], especially atrial longitudinal strain, as a good parameter for the detection of LA function in different conditions such as AF. STE and LA strain are very good tools for LA function assessment [12].

Several studies have established the association between POAF in CAD patients and LA dysfunction using TDI and 2D speckle tracking with its angle independence and the ability to specify myocardial active motion and passive tethering.

In our study, the postoperative AF was 31% (28 patients of 89 patients). *EXCEL trial* demonstrated that new-onset AF had an 18% rate after CABG [13]. Several studies demonstrated that postoperative AF was observed post-CABG with a frequency of 20–40% [14].

This study showed no difference in general demography between the two groups except for age as the POAF group was significantly older which is consistent with Nevzat Erdil et al. [15], Mariscalco et al. [11], Mahoney et al. [16], and Hernandez et al. [17] findings. The POAF was similar in both genders which is consistent with Lee et al. [18] and in contrast to Mahoney et al. [16] who stated that male gender had a higher incidence of POAF.

Hernandez et al. [17] meta-analysis revealed that obesity had a moderate risk of developing POAF while in our study body mass index and obesity showed no statistical value. Sun et al. [19] found a relation between high BMI and incidence of POAF with an incidence of 50% of occurrence in BMI 35–40 (severely obese), but they found no correlation between normal-overweight BMI and POAF which is consistent with our study (mean BMI in our study is 32.0 ± 3.8).

Regarding other risk factors, both groups were the same regarding hypertension, diabetes, or smoking with no statistical significance which is consistent with what Nevzat Erdil et al. [15] concluded after performing 1040 isolated CABG surgeries and following up for POAF, in contrast to what Kalus et al. [2] found in their sub-study indicating that DM and postoperative use of NSAIDs predict the occurrence of POAF.

Regarding the preoperative data, both groups were nearly equal regarding NYHA class, Canadian classification, number of vessels affected, and preoperative medications which is consistent with Nevzat Erdil et al. [15] and Luigi Gabrielli et al. [20] findings.

Surprisingly, the administration of preoperative nitrates showed a better outcome on the occurrence of POAF. 91.8% of the no POAF group were on preoperative nitrates while only 57.1% of the POAF group were on preoperative nitrates. The preoperative nitrate administration was not studied before, while the postoperative nitrate administration with the inotropes and antiplatelets showed an increased risk for POAF as showed by Efird et al. [21].

Regarding the intraoperative data, both groups showed no statistical significance regarding the time cross-clamp, bypass duration, and number of grafts which is consistent with Nevzat Erdil et al. [15] results. These results were contradicted by an Indian study by Dave et al. [22] conducted on 150 patients and showed that patients with cross-clamp duration of more than 1 h and bypass duration of more than 100 min showed a statistically significant higher value of POAF. But these results might have been different on a larger scale like Nevzat Erdil et al. [15] (1040 patients).

In the postoperative period, the POAF group showed a higher incidence for stroke (14.3% vs 3.3% in the no POAF group) and a longer hospital stay (11.5 ± 1.8 vs 10.4 ± 2.3 in no POAF) in agreement with Nevzat Erdil et al. [15] and Echahidi et al. [23] who report that POAF lengthens hospital stay by 4.9 days mostly due to these patients had attempts to restore sinus or control of heart rate and begin and control of anticoagulants beside association between AF and other morbidities [23].

The postoperative use of inotropes did not increase the incidence of POAF in this study, contrary to Efird et al. [21] findings. They deduced that patients on peri-operative inotropic medications have a higher incidence of POAF especially when adding nitrates and antiplatelets. He built his hypothesis on the assumption that nitrates if added to antiplatelet decrease the formation of guanosine monophosphate, so increases free radicals, catecholamines, and plasma volume.

In the preoperative echocardiographic data, both groups showed similar left ventricular end-diastolic, end-systolic diameters and ejection fraction. The left atrial diameters were similar in both groups in contrast to the findings of Luigi Gabrielli [20], who found that POAF has larger left atrial diameters.

The peak atrial longitudinal strain (PALS) data were consistent with Luigi Gabrielli [20], Hirose et al. [7], Verdejo et al. [22], and Her et al. [24] studies. All of these studies suggested that PALS is highly significant in predicting POAF. The strain rate data had some controversies; our study showed that the strain rate values showed statistical difference with a lower systolic strain rate (LASR) and higher (less negative) early LASRe and late LASRa diastolic strain rates, while Luigi Gabrielli, [20], Hirose et al. [7], and Verdejo et al. [22] results showed the significance of the systolic strain rate (LASRs) and late LASRa diastolic strain rate only.

In our study, the POAF patients showed significantly lower peak atrial longitudinal strain value than non-POAF patients (25.6 ± 6.1 vs 32.9 ± 5.9). The left atrial strain rate values showed significant difference with lower left atrial systolic strain rate (1.27 ± 0.56 vs 1.9 ± 0.71) and less negative (higher) early diastolic strain rate (−1.0 ± 0.45 vs −1.69 ± 0.6) and late diastolic strain rate (−1.21 ± 0.48 vs −2.21 ± 0.59).

After multivariate analysis in our study, the independent predictors for POAF were peak atrial longitudinal strain (PALS) (OR 0.770, 95% CI 0.627–0.946), LASRa (OR 3.476, 95% CI 1.207–12.186), and age (OR 1.181, 95% CI 1.011–1.379). In contrast to Luigi Gabrielli et al. [20] who showed in their study that the independent predictors for POAF were age, LASRs, and LASRa [20], Hirose et al. [7] and Verdejo et al. [22] both found that age and PALS were the predictors of POAF.

So PALS was found to be a simple, reproducible, single step, and easily detected predictor of the occurrence of POAF after CABG.

## Conclusion

- ▪ Multiple predictors can be used for the detection of POAF, e.g., age.
- ▪ New-onset AF post-CABG is linked to LA dysfunction when assessed by preoperative 2D speckle tracking echocardiography.
- ▪ Strain and strain rate showed to be an applicable method for the prediction of POAF, and due to the technical difficulties of the strain rate measurements, speckle tracking-derived strain curve shows to be a simple and single-step approach to the detection of the POAF.
- ▪ PALS of less than 29.8 could be suggested as a cut-off value for the occurrence of POAF, but it needs further studies with larger numbers.

## Study limitations

- The small sample size and short period of follow-up.
- As the study is based on echocardiography, so image quality and operator experience have a great influence on the results.
- The software of the left ventricle had been used for the analysis of LA strain.
- Continuous monitoring was done only in the critical care unit and in the postoperative ward; ECG was done every 12 h or symptoms appear. So any undetected or transient episode of AF could be missed.
- Postoperative LA function could not be assessed as it is difficult to obtain good images for analysis.
- The accuracy of LA strain was not compared with the invasive assessment method or non-invasive method such as CMR.

## Data Availability

The data sets used and/or analyzed during the current study are available from the corresponding author on reasonable request.

## Abbreviations

2D: 2-Dimensional
AF: Atrial fibrillation
ASE: American Society of Echocardiography
BMI: Body mass index
CABG: Coronary artery bypass grafting
CAD: Coronary artery disease
CMR: Cardiac magnetic resonance imaging
COPD: Chronic obstructive airway disease
DM: Diabetes mellitus
DT: Deceleration time
LA: Left atrium
LAS: Left atrial strain
PALS: Peak atrial longitudinal strain
STE: Speckle tracking echocardiography
